# Clinical Utility of Serum sCD200/sCD200R Ratios in Predicting Current Activity of Antineutrophil Cytoplasmic Antibody-Associated Vasculitis

**DOI:** 10.3390/jcm14082720

**Published:** 2025-04-15

**Authors:** Jang Woo Ha, Jihye Chung, Taejun Yoon, Yong-Beom Park, Sang-Won Lee

**Affiliations:** 1 Division of Rheumatology, Department of Internal Medicine, Yongin Severance Hospital, Yonsei University College of Medicine, Yongin 16995, Gyeonggi-do, Republic of Korea; hjwnmk@yuhs.ac; 2 Division of Rheumatology, Department of Internal Medicine, Yonsei University College of Medicine, Seoul 03722, Republic of Korea; 3 Institute for Immunology and Immunological Diseases, Yonsei University College of Medicine, Seoul 03722, Republic of Korea

**Keywords:** CD200, CD200R, antineutrophil cytoplasmic antibody-associated vasculitis, activity

## Abstract

**Objectives**: This study investigated whether serum soluble CD200 (sCD200) and soluble receptor for CD200 (sCD200R) concentrations and serum sCD200/sCD200R ratios at diagnosis could predict cross-sectional activity in patients with antineutrophil cytoplasmic antibody (ANCA)-associated vasculitis (AAV). **Methods**: We included 70 patients with AAV in this pilot study, retrospectively reviewed their medical records, and collected clinical data at the time of AAV diagnosis. We also measured sCD200 and sCD200R in stored blood samples collected at diagnosis. In medical records, AAV activity at diagnosis had been assessed according to the Birmingham Vasculitis Activity Score (BVAS). The prediction potential of serum sCD200 and sCD200R concentrations and serum sCD200/sCD200R ratios for BVAS was evaluated using Pearson correlation analysis. **Results**: Among the 70 patients, the median age was 63.5 years, with 29 males and 41 females. Among the three CD200-related variables at diagnosis, serum sCD200/sCD200R ratios at diagnosis were significantly correlated with cross-sectional BVAS; however, serum sCD200 and sCD200R concentrations were not correlated with it. These results may indicate that serum sCD200/sCD200R ratios may better help predict cross-sectional AAV activity by increasing the range of opposing changes in the two variables. On the other hand, both serum sCD200 concentrations and serum sCD200/sCD200R ratios showed significant correlations with cross-sectional myeloperoxidase-ANCA titre, five-factor score, and serum creatinine levels at diagnosis. **Conclusions**: In this study, we demonstrated that serum sCD200/sCD200R ratios at diagnosis can be a useful and convenient biomarker to predict cross-sectional AAV activity calculated according to BVAS.

## 1. Introduction

CD200, a 41–47 kDa glycoprotein, is a Type-I transmembrane glycoprotein belonging to the immunoglobulin superfamily and is composed of three domains, including extra-cellular, transmembrane, and short cytoplasmic domains [1,2]. The interaction between CD200 and CD200R, the receptor for CD200, can induce the inhibitory signalling pathway. When CD200 binds to CD200R, the activity of immune cells may be modulated and further, their responses to inflammatory signals may be suppressed [3,4]. Additionally, the soluble forms of CD200 (sCD200) and CD200R (sCD200R) can be produced and released by cytoplasmic cleaving enzymes [5,6]. Regarding sCD200, the inducers or stimulators for sCD200 production have not been clarified. However, serum sCD200 concentrations have been considered to be associated with the current extent of inflammatory burden including serum interleukin (IL)-6 levels, or the present conditions of chronic inflammatory diseases or haematological malignancies [7,8]. Regarding sCD200R, it could be theoretically assumed that as both membrane-bound CD200, and sCD200 production increases, both CD200R and sCD200R production may be reduced through the inhibitory signalling pathway [3,4]. Additionally, given the previous report that sCD200 could bind to membrane-bound CD200R [6]; thus, it can be assumed that there may be an inverse relationship between sCD200 and sCD200R production and serum concentrations. Therefore, the ratios of serum sCD200 and sCD200R concentrations (sCD200/sCD200R ratios) could also be considered a biomarker as good as serum sCD200 concentrations in several chronic inflammatory diseases.

Antineutrophil cytoplasmic antibody (ANCA)-associated vasculitis (AAV) is a form of small-vessel vasculitis characterised by fibrinoid necrotizing inflammation primarily involving capillaries, venules, and arterioles, and occasionally extending to medium-sized arteries. As the name suggests, ANCAs play critical roles in the pathogenesis of AAV. AAV has three subtypes according to the prevailing clinical features such as microscopic polyangiitis (MPA), granulomatosis with polyangiitis (GPA), and eosinophilic granulomatosis with polyangiitis (EGPA). MPA primarily involves the lungs and kidneys, which may provoke diffuse alveolar haemorrhage and glomerulonephritis. In contrast, GPA frequently involves both the upper and lower respiratory tracts including refractory retro-orbital lesions, otitis media, sinusitis, subglottic or bronchial stenosis, and pulmonary nodules and cavitation. On the other hand, EGPA has prodromal, eosinophilic, and vasculitic phases, which may exhibit both allergic and vasculitic clinical features [9,10].

Estimating the cross-sectional activity of AAV is important for selecting therapeutic strategies and anticipating and coping with future poor outcomes. Currently, the Birmingham vasculitis activity score (BVAS) is a widely used and validated tool for assessing disease activity in patients with AAV. BVAS consists of nine systemic items and each item has several subitems. A differently weighted score is assigned to each subitem and, depending on whether it is persistent or new/worsening, the score is given differently. A maximum persistent score is 33, whereas a maximum new/worsening score is up to 63 [11]. However, BVAS has several issues: for instance, it takes a long time to fill in the BVAS form because the clinical manifestations within 4 weeks before the assessment should be reviewed [11]. Therefore, a need for serum biomarkers to estimate the cross-sectional AAV has been raised; however, no immediate and convenient serum biomarkers to predict the current activity of AAV based on BVAS have been established till now. Given that CD200, the source of sCD200, is mainly expressed in activated T and B cells that play an important role in AAV pathophysiology [1,2], and is also expressed in endothelial cells [12], it could be reasonably speculated that serum sCD200 and sCD200R concentrations would be biomarkers for predicting current inflammatory status at diagnosis in patients with AAV. Nevertheless, until now, there has been no study investigating the utility of serum sCD200 and sCD200R concentrations in predicting current activity in patients with AAV. Therefore, this study aimed to determine whether serum sCD200 and sCD200R concentrations and serum sCD200/sCD200R ratios at diagnosis, are predictive of cross-sectional disease activity in patients with AAV.

## 2. Materials and Methods

### 2.1. Study Subjects

We retrospectively reviewed the electronic medical charts of 70 patients who were arbitrarily selected from the single-centre cohort of Korean patients with AAV in this hospital. Patients included in this study were newly diagnosed with AAV at the Department of Internal Medicine, Yonsei University College of Medicine, Severance Hospital, between November 2005 and December 2023.They needed to meet both the revised nomenclature of systemic vasculitides proposed by the Chapel Hill Consensus Conference in 2012 and the algorithm for the classification of AAV suggested by the European Medicine Agency in 2007 [9,10]. They needed to be reclassified as having AAV according to the new classification criteria for AAV proposed by a joint group of the American College of Rheumatology (ACR), and the European Alliance of Association for Rheumatology (EULAR) in 2022 [13,14,15,16]. Sufficient clinical, laboratory, radiological, and histological information at diagnosis and during follow-up until the last visit was required in the electronic medical records for AAV classification. Only ANCA test results collected within 2 weeks before or after AAV diagnosis were approved. Additionally, patients had no coexisting serious medical conditions that could mimic AAV, such as malignancies or severe infectious disease [13,14,15]. They had never received immunosuppressive drugs within 4 weeks before AAV diagnosis. They needed to give consent upon providing blood samples and clinical data documented at diagnosis.

### 2.2. Ethics Approval

The present study was approved by the Institutional Review Board (IRB) of Severance Hospital, Seoul, Republic of Korea (IRB number 4-2016-0901) on 5 December 2016. All patients had previously provided written informed consent at the time of AAV diagnosis for the use of blood samples and clinical data. The IRB waived the requirement for additional consent.

### 2.3. Clinical Data

At the time of AAV diagnosis, demographic variables including age, sex, smoking history, and body mass index were recorded. Clinical data collected included AAV subtypes, ANCA positivity and titres, as well as AAV-specific indices such as BVAS and the Five-Factor Score (FFS) [11,17]. Particularly, we retrospectively collected BVAS and FFS, which were assessed and documented at the time of AAV enrolment in the cohort of AAV. In accordance with the 2022 ACR/EULAR classification criteria for AAV, ANCA test results included both perinuclear (P)-ANCA and cytoplasmic (C)-ANCA detected by indirect immunofluorescence assay, as well as MPO-ANCA and PR3-ANCA measured by immunoassay [13,14,15,16]. Only type 2 diabetes mellitus, hypertension, and dyslipidaemia, which occurred before AAV diagnosis, were recognised as initial comorbidities [18]. Erythrocyte sedimentation rate (ESR) and C-reactive protein (CRP) were assessed as markers of acute-phase inflammation. [19]. The remaining laboratory variables at diagnosis are described in Table 1.

### 2.4. sCD200 and sCD200R Measurement

On the day of written informed consent, whole blood was collected, and serum was promptly isolated and stored at −80 °C for subsequent analysis. Serum sCD200 and sCD200R concentrations were measured from the stored sera using enzyme-linked immunosorbent assay kits (Abcam, Cambridge, UK) according to the protocol of the manufacturers. The serum sample analyses were conducted in duplicates to minimise intra-assay variability.

### 2.5. Statistical Analyses

All statistical analyses were performed using SPSS version 26 (IBM Corporation, Armonk, NY, USA) for Windows (Microsoft Corporation, Redmond, WA, USA). Continuous variables were expressed as medians with interquartile ranges (Q1 to Q3), and categorical variables as numbers with percentages. Correlation between two variables was assessed using Pearson’s correlation coefficient (r). A *p* value < 0.05 was considered to be statistically significant.

## 3. Results

### 3.1. Characteristics of Patients at Diagnosis

Among the 70 patients included in this study, the median age was 63.5 years, with 29 males and 41 females. Three patients were ex-smokers, and the median body mass index was calculated as 22.2 kg/m2. Regarding AAV subtypes, 33 patients were diagnosed with MPA, 20 with GPA, and 17 with EGPA. Thirty-seven tested positive for MPO-ANCA (or P-ANCA) and eleven for PR3-ANCA (or C-ANCA). The median BVAS, FFS, ESR, and CRP were 7.5, 0, 23.0 mm/h, and 3.8 mg/L, respectively. The median serum sCD200 and sCD200R concentrations, and sCD200/sCD200R ratios were 253.6 pg/mL, 2799.0 pg/mL, and 0.08, respectively. The remaining laboratory results are described in Table 1.

### 3.2. Correlation Analyses of Three CD200-Related Variables at Diagnosis with Cross-Sectional BVAS

**Figure 1:**
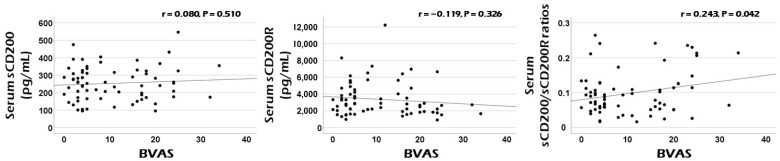
Correlation analyses. Among three serum sCD200-related variables, only serum sCD200/sCD200R ratios exhibited a clinical potential for estimating the cross-sectional AAV activity represented by BVAS at diagnosis in patients with AAV. BVAS: the Birmingham vasculitis activity score; s: soluble; R: receptor; ANCA: antineutrophil cytoplasmic antibody; AAV: ANCA-associated vasculitis.

Among three CD200-related variables at diagnosis, only serum sCD200/sCD200R ratios at diagnosis were significantly correlated with cross-sectional BVAS in patients with AAV. Neither serum sCD200 nor sCD200R concentrations were remarkably correlated with cross-sectional BVAS (Figure 1).

### 3.3. Correlation Analyses of Three CD200-Related Variables at Diagnosis with the Remaining Cross-Sectional Continuous Variables

Serum sCD200 concentrations were significantly correlated with age, MPO-ANCA titre, FFS, platelet count, and serum creatinine levels at diagnosis; however, serum sCD200R concentrations exhibited no correlation with continuous variables at diagnosis. Additionally, serum sCD200/sCD200R ratios were significantly correlated with MPO-ANCA titre, FFS, ESR, haemoglobin, and serum creatinine levels at diagnosis, in addition to BVAS. Meanwhile, MPO-ANCA titre, FFS, and serum creatinine levels showed significant correlations with two of the three CD200-related variables, both serum sCD200 concentrations and serum sCD200/sCD200R ratios (Table 2).

## 4. Discussion

It has been reported that sCD200 and sCD200R production and release have great clinical potential to reflect the extent of inflammation in several chronic inflammatory diseases and malignancies [7,8]. Therefore, in this study, we investigated whether serum sCD200 and sCD200R concentrations and serum sCD200/sCD200R ratios at diagnosis could predict cross-sectional activity in patients with AAV. We obtained several interesting findings. First, serum sCD200/sCD200 ratios at diagnosis were significantly correlated with cross-sectional BVAS, whereas the serum concentrations of the two corresponding parameters were not. Second, both serum sCD200 concentrations and serum sCD200/sCD200R ratios showed significant correlations with cross-sectional MPO-ANCA titre, FFS, and serum creatinine levels at diagnosis. Therefore, we suggest the possibility that serum sCD200/sCD200R ratios at diagnosis can be a useful and convenient biomarker to predict cross-sectional AAV activity calculated according to BVAS.

**Figure 2:**
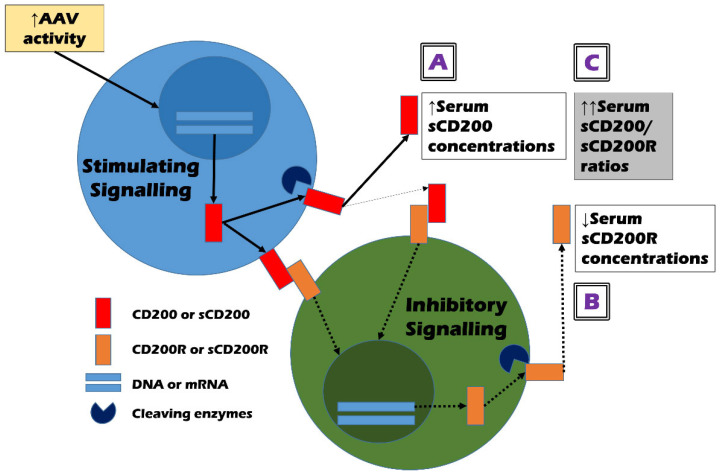
Hypotheses on the mechanism of how serum sCD200/sCD200R ratios could estimate the cross-sectional activity of AAV. (**A**) high AAV activity may increase the production of sCD200; (**B**) high AAV activity may inhibit the production of sCD200R; and (**C**) thus, high AAV activity may amplify the ratios of sCD200 and sCD200R by increasing their differences. Solid arrows and dotted arrows represent stimulatory, and inhibitory signal transmissions, respectively. ANCA: antineutrophil cytoplasmic antibody; AAV: ANCA-associated vasculitis; s: soluble; R: receptor; DNA: deoxyribonucleic acid; mRNA: messenger ribonucleic acid.

The direct immunologic role of CD200 and CD200R in the pathogenesis of AAV is unclear. However, given that both CD200 and CD200R are mainly expressed in activated T and B cells in addition to various cell types [1,2], we tried to explain the mechanism of how serum sCD200 and sCD200R concentrations could predict cross-sectional AAV activity and came up with several hypotheses. First, in a microenvironment where AAV activity is increased, various inflammation-related substances may turn on the stimulating signalling pathway and promote CD200 expression and production [1,7,8]. Membrane-bound CD200 is cleaved by cleaving enzymes to release only the extracellular domain and generate sCD200 [5,6]. At this time, the higher the stimulus caused by AAV activation increases, the more CD200 may be produced: the total number of both membrane-bound CD200 and sCD200 can increase. Therefore, the first hypothesis is that high AAV activity can induce an increase in serum sCD200 concentrations (Figure 2A). Next, once membrane-bound CD200 binds to CD200R expressed on the surfaces of the counteracting cells; this binding may turn on the inhibitory signalling pathway. Also, it may diminish CD200R production in addition to leading to a decrease in the production of inflammation-related cytokines and similar substances [3,4]. Similarly to the process of sCD200 production, sCD200R can be generated by cleaving enzymes and its amount may be positively proportional to the total amount of CD200R production. Moreover, sCD200 can also bind to membrane-bound CD200R and accelerate this series of inhibitory signalling processes [6]. Therefore, the second hypothesis is that high AAV activity can increase both membrane-bound CD200 and sCD200, which in turn, can decrease the total CD200R production, leading to a decrease in serum sCD200R concentrations (Figure 2B).

In summary, the total production of CD200 and CD200R may be regulated by current AAV activity and further, may form a complementary (or negative) feedback loop in a close interrelationship. Therefore, both serum sCD200 concentrations and serum sCD200R concentrations have sufficient potential as good biomarkers that can reflect and predict cross-sectional AAV activity. However, the following situations cannot be ignored. The increase in total CD200 production may not directly predict the decrease in total CD200R production. In other words, the absolute values of the increase in total CD200 production and the decrease in total CD200R production may not necessarily be proportional. Additionally, the activity of cleaving enzymes that produce sCD200 and sCD200R may be important confounding variables. Given these situations, the third hypothesis is that serum sCD200/sCD200R ratios may better help estimate cross-sectional AAV activity by increasing the range of opposing changes in the two variables (Figure 2C). In this study, although serum sCD200 concentrations did not flexibly reflect the change range of cross-sectional BVAS as much as FFS or serum creatinine, serum sCD200/sCD200R ratios showed a statistically significant positive correlation with cross-sectional BVAS as efficiently as FFS and serum creatinine (Figure 1 and Table 2). This indicates the relative clinical superiority among the three CD200-related soluble forms and supports our last hypothesis.

The present study has the advantage that it is the first to demonstrate that serum sCD200/sCD200R ratios at AAV diagnosis could predict cross-sectional AAV activity. Moreover, the additional advantage is that we proposed several hypotheses on the mechanisms of how they could be clinically useful in patients with AAV. However, because this study was only conducted on Korean patients with AAV, it may not be appropriate to immediately apply the results of this study to patients with other geography or ethnicity. Therefore, this study has another advantage in that it provides the method to select one of the three sCD200-related variables and validate it as a biomarker for estimating AAV activity. This study also has some limitations. Because this study is a pilot study exploring the clinical potential of sCD200 and sCD200R, the sample size was not large enough to allow for the generalisation of this study’s findings. Also, because this study analysed clinical data retrospectively and measured serum sCD200 and sCD200R concentrations using stored blood collected at AAV diagnosis, it was impossible to completely overcome the inherent limitations of retrospective research.

However, as a pilot study, our findings offer a foundation for future research. A larger-scale study involving more patients with AAV and serially collected paired blood samples is warranted to further validate and expand on the clinical utility of serum sCD200 concentrations and serum sCD200/sCD200R ratios at the time of diagnosis in patients who are newly diagnosed with AAV.

## 5. Conclusions

This study is the first to show that the serum sCD200/sCD200R ratio at diagnosis can be a useful and convenient biomarker to predict cross-sectional AAV activity calculated according to BVAS. Therefore, we suggest that serum sCD200 and sCD200R be measured and that their ratios be calculated in patients newly diagnosed with AAV, as a complementary biomarker possessing the clinical potential to reflect the extent of the inflammatory burden by directly or indirectly considering the inhibitory co-signals of the immune cells involved in the pathogenesis of AAV.

## Figures and Tables

**Table 1:** Characteristics of patients with AAV at diagnosis (N = 70).

Variables	Values
Demographic data	
Age (years)	63.5 (52.8–73.3)
Male sex (N, (%))	29 (41.4)
Female sex (N, (%))	41 (58.6)
Ex-smoker (N, (%))	3 (4.3)
Body mass index (kg/m^2^)	22.2 (20.6–24.7)
AAV subtypes (N, (%))	
MPA	33 (47.1)
GPA	20 (28.6)
EGPA	17 (24.3)
ANCA titres and positivity (N, (%))	
MPO-ANCA titre	0 (0–37.3)
PR3-ANCA titre	0 (0–0)
MPO-ANCA (or P-ANCA) positive	37 (52.9)
PR3-ANCA (or C-ANCA) positive	11 (15.7)
MPO-ANCA (P-ANCA) as well as PR3-ANCA (C-ANCA) positive	2 (2.9)
ANCA negative	24 (34.3)
AAV-specific indices	
BVAS	7.5 (3.0–17.3)
FFS	0 (0–1.0)
Comorbidities (N, (%))	
Type 2 diabetes mellitus	17 (24.3)
Hypertension	23 (32.9)
Dyslipidaemia	12 (17.1)
Acute-phase reactants	
ESR (mm/hr)	23.0 (7.0–85.0)
CRP (mg/L)	3.8 (0.9–31.8)
Laboratory results	
White blood cell count (/mm^3^)	7850.0 (6175.0–11,062.5)
Haemoglobin (g/dL)	12.2 (10.2–13.7)
Platelet count (×1000/mm^3^)	248.0 (190.0–367.0)
Serum creatinine (mg/dL)	0.8 (0.6–1.5)
Serum albumin (g/dL)	4.2 (3.5–4.4)
Serum sCD200-related variables	
Serum sCD200 (pg/mL)	253.6 (173.8–318.4)
Serum sCD200R (pg/mL)	2799.0 (1918.8–4295.3)
Serum sCD200/sCD200R ratios	0.08 (0.06–0.13)

Values are expressed as a median (25~75 percentile) or N (%). ANCA: antineutrophil cytoplasmic antibody; AAV: ANCA-associated vasculitis; MPA: microscopic polyangiitis; GPA: granulomatosis with polyangiitis; MPO: myeloperoxidase; PR3: proteinase 3; P: perinuclear; C: cytoplasmic; BVAS: the Birmingham vasculitis activity score; FFS: the five-factor score; ESR: erythrocyte sedimentation rate; CRP: C-reactive protein; s: soluble; R: receptor.

**Table 2:** Correlation analysis of serum sCD200 and sCD200R concentrations, and serum sCD200/sCD200R ratios with continuous variables at diagnosis in patients with AAV.

	Serum sCD200 Concentrations		Serum sCD200R Concentrations		Serum sCD200/sCD200R Ratios	
	Correlation Coefficient (r)	*p* Value	Correlation Coefficient (r)	*p* Value	Correlation Coefficient (r)	*p* Value
Demographic data						
Age	0.374	0.001	0.005	0.966	0.224	0.063
Body mass index	0.041	0.734	0.100	0.408	−0.089	0.464
ANCA titres						
MPO-ANCA titre	0.260	0.030	0.008	0.945	0.251	0.036
PR3-ANCA titre	−0.180	0.137	−0.111	0.362	−0.074	0.542
AAV-specific indices						
BVAS	0.080	0.510	−0.119	0.326	0.243	0.042
FFS	0.237	0.048	−0.165	0.173	0.358	0.002
Acute-phase reactants						
ESR	−0.077	0.551	0.210	0.098	−0.251	0.048
CRP	0.130	0.295	0.052	0.676	0.078	0.528
Laboratory results						
White blood cell count	−0.183	0.130	−0.194	0.107	0.057	0.638
Haemoglobin	−0.092	0.448	0.219	0.069	−0.268	0.025
Platelet count	−0.294	0.014	−0.102	0.403	−0.148	0.223
Serum creatinine	0.396	0.001	−0.173	0.151	0.389	0.001
Serum albumin	−0.125	0.309	0.156	0.205	−0.205	0.093

s: soluble; R: receptor; ANCA: antineutrophil cytoplasmic antibody; AAV: ANCA-associated vasculitis; MPO: myeloperoxidase; PR3: proteinase 3; BVAS: the Birmingham vasculitis activity score; FFS: the five-factor score; ESR: erythrocyte sedimentation rate; CRP: C-reactive protein.

## Data Availability

The data used to support the findings of this study are included within the article and the Supplementary Materials.

## Supplementary Materials

The following are available online at https://www.mdpi.com/article/10.3390/jcm14082720/s1, Table S1: Clinical manifestations based on the systemic items of BVAS at diagnosis.
