# Supplementary material for: Clinical Utility of Serum sCD200/sCD200R Ratios in Predicting Current Activity of Antineutrophil Cytoplasmic Antibody-Associated Vasculitis
Source: J Clin Med. 2025 Apr 15;14(8):2720. doi: 10.3390/jcm14082720 (PMC12027553; doi:10.3390/jcm14082720)
Supplement: Supplementary file 1 [file jcm-14-02720-s001.zip › jcm-3554560-supplementary.pdf]

Supplementary Table S1. Clinical manifestations based on the systemic items of BVAS at diagnosis (N=70).

Variables	Values
**Systemic major organ involvement (N, (%))**	
General manifestations	16 (22.9)
Cutaneous manifestations	11 (15.7)
Mucous and ocular manifestations	6 (8.6)
Ear nose and throat manifestations	39 (55.7)
Pulmonary manifestations	48 (68.6)
Cardiovascular manifestations	8 (11.4)
Gastrointestinal manifestations	0 (0)
Renal manifestations	35 (50.0)
Proteinuria*	27 (38.6)
Haematuria**	16 (22.9)
Nervous systemic manifestations	26 (37.1))

BVAS: the Birmingham vasculitis activity score.
*Proteinuria: ≥+2 on urine stick.
**Haematuria: ≥10 RBCs on high power field.
